# Protein landscape of the brush border membrane of first instar larvae of *Frankliniella occidentalis*, the western flower thrips

**DOI:** 10.1371/journal.pone.0326260

**Published:** 2025-06-24

**Authors:** Marlonni Maurastoni, Dorith Rotenberg, Ruchir Mishra, Bryony C. Bonning, Anna E. Whitfield

**Affiliations:** 1 Department of Entomology and Plant Pathology, North Carolina State University, Raleigh, North Carolina, United States of America; 2 Department of Entomology and Nematology, University of Florida, Gainesville, Florida, United States of America; University of Florida Tropical Research and Education Center, UNITED STATES OF AMERICA

## Abstract

We investigated the protein composition of the brush border membrane of larval *Frankliniella occidentalis* (western flower thrips), an agriculturally significant crop pest and vector of plant pathogens. We developed a protocol for purifying brush border membrane vesicles (BBMVs) from first-instar larvae (L1) bodies and identified their protein composition by LC-MS/MS. From 2544 proteins identified, 469 were predicted to be secreted and part of the inner and outer plasma membrane leaflet using cell-localization prediction tools and homology with reviewed proteins in the UniProt database. Comparison to thrips tissue-specific proteomes revealed that 371 and 263 of the identified BBMV plasma membrane and secreted proteins matched the larval gut and adult salivary glands, respectively. Annotations of most of the proteins inferred ‘catalytic activity’ (56.3%) and ‘binding’ (49.6%), with an overrepresentation of proteins involved in protein digestion, specifically serine proteases, lipid transport, and ATPase activity. Bioinformatic-enabled comparisons to thrips tissue-specific proteomes and transcriptomes enabled us to predict the secretome and the plasma membrane proteins of larval thrips’ gut epithelial cells, providing new targets for thrips control.

## Introduction

*Frankliniella occidentalis* (order Thysanoptera, suborder Terebrantia, family Thripidae), commonly known as the western flower thrips, is an invasive pest with a worldwide distribution, large plant host range (highly polyphagous), and propensity for rapid development of insecticide resistance (reviewed in [1]). As such, it threatens agricultural production and challenges pest management strategies. *Frankliniella occidentalis* primarily feeds on plant fluids by puncturing, sucking and ingesting the contents of individual plant cells, causing significant damage to a wide variety of vegetable, fruit and ornamental crops. Their feeding habit results in direct damage, such as stunted growth, deformed leaves and fruits, and fruit scars and blemishes, rendering the produce unmarketable. Relatively few thrips species (subfamily Thripinae), including *F. occidentalis*, are also notorious for their role as vectors of plant-pathogenic viruses (notably, orthotospoviruses) to diverse plant hosts, leading to extensive crop losses [2].

The brush border is a specialized structure on the apical surface of epithelial cells, characterized by densely packed microvilli, which are cellular finger-like membrane projections structurally sustained by a core of actin filaments. These microvilli significantly increase the surface area of the cell, aiding in the absorption and digestion of nutrients [3] and the secretion of substances [4,5]. They vary significantly among different organs in both cytoskeletal and membrane protein compositions, reflecting their specialized functions [4]. These structures are fundamental to the efficient functioning of various insect anatomical structures such as the gut, salivary glands, and Malpighian tubules [5]. In the gut, microvilli extend the epithelial surface, facilitating the digestion and absorption of nutrients, crucial for meeting the energy requirements of the insect. Similarly, in the salivary glands, microvilli assist in the secretion of enzymes and other substances that aid in food processing. In Malpighian tubules, the primary excretory and osmoregulatory organs in insects, microvilli play a vital role in the reabsorption of solutes and water, thereby maintaining internal homeostasis [6].

Early investigations of the internal anatomy of *F. occidentalis* adults at the ultrastructural level revealed significant features of the digestive system, particularly relating to brush border membranes and surrounding, lumen-facing perimicrovillar membrane characteristic of paraneopteran midguts [7–11]. The midgut of *F. occidentalis* is composed of three loops, each formed by a single layer of columnar epithelial cells. These cells are lined with numerous microvilli in the gut lumen, with the cytoplasm within the villi oriented along the axis exhibiting a filamentous appearance like fine fibrils. According to Ullman [9], this arrangement in thrips – not observed in the microvillar brush border of hindgut and Malpighian tubules – likely facilitates substrate transport from the gut lumen into the epithelial cells, thereby enhancing nutrient absorption and digestion. While no consensus has been reached on the presence of a glycocalyx with fork-like structures protruding from the microvilli of the posterior midgut, the presence of a structure that resembles a perimicrovillar membrane has been observed in the anterior part of the adult midgut. This structure encloses one or several microvilli in a bundle, along with droplets of dense material [7,8]. Additionally, at the junction of the midgut and the hindgut, three Malpighian tubules are found, each also characterized by a single layer of epithelial cells with a brush border composed of tightly packed microvilli [9]. At the distal region of the tubular salivary glands a microvilli is also observed, with a cell apex composed of a short-curved microvilli, interdigitated in an orderly way to form a compact layer [8].

Brush border membrane vesicles (BBMVs) are microscopic, spherical vesicles derived from the brush border membrane of epithelial cells, particularly those lining the gut. Purification of BBMVs is important for studying membrane proteins and enzymes involved in nutrient absorption and digestion, providing insights into gut physiology. BBMVs have been used for disease research, particularly for understanding pathogen interactions with the gut epithelium [12], a key site for infection and drug absorption. Additionally, BBMVs have proven to be useful tools for studying the function and regulation of membrane transporters and other membrane-associated proteins, and as such, are valuable tools in toxicological studies to investigate drug binding and uptake mechanisms. Despite the importance of insect gut proteins in a range of biological processes, few studies have characterized the composition and abundance of proteins on the gut brush border membrane of insects. Major challenges to overcome in studying the insect gut brush border membrane proteome are separating brush border membrane proteins from other gut components and identifying low-abundance proteins that may be critical for insect-microbial interaction. In general, BBMVs are prepared through a series of meticulous laboratory procedures that utilize calcium or magnesium chloride to induce the formation of vesicles from the brush border membranes. Proteomes of BBMVs enriched in insect gut plasma membrane proteins have been described for mosquitoes [13,14], lepidopterans [15–18], and hemipterans, namely the Asian citrus psyllid, *Diaphorina citri*, the vector of plant-pathogenic bacteria of the *Candidatus* Liberibacter genus [19], the green peach aphid, *Myzus persicae*, as well as the whitefly *Bemisia tabaci* [20]. Neither the gut brush border proteome nor BBMV-isolation has been previously reported for any thysanopteran (i.e., thrips).

A better understanding of the gut brush border membrane proteome of thrips could provide critical insights into the molecular mechanisms underlying digestion, insecticide detoxification and microbial and/or viral interactions. We report herein a detailed characterization of the composition and abundance of the proteins present in larval thrips BBMVs. We used BBMVs to determine the protein profile of the gut brush border membrane. We selected larval instars because they are not only voracious consumers compared to adults, but young *F. occidentalis* larval midguts are the first barrier to internalization of orthotospoviruses into the vector body [2]. We developed a protocol for the preparation of BBMVs from thrips whole body first instars, requiring copious numbers of pooled larvae due to their minute size and total protein concentration needed for robust BBMV proteome analysis. Proteins in BBMV samples were identified through LC-MS/MS and their localization on the plasma membrane was predicted using bioinformatic tools and comparisons to an *F. occidentalis* larval gut protein sequence database [21]. We discuss the inferred physiological processes on the thrips gut brush border membrane from the known functions and roles of these identified proteins in other biological systems.

## Materials and methods

### Insects

The *F. occidentalis* colony was originally started using insects gathered from Kamilo Iki Valley, Oahu, Hawaii, and reared on green bean (*Phaseolus vulgaris*) pods under laboratory conditions [22]. This particular isolate has been in colony under our care for approximately 20 years, reared and age-structured on green bean pods at laboratory ambient room temperature (22°C – 23°C) and relative humidity (47% − 53%), with supplemental fluorescent growth lights (14 h light: 10 h dark cycle). Insects were synchronized based on their developmental stages to ensure they were of the same age at time of sampling.

### BBMV preparation from thrips first instar larvae

Age-synchronized first instar larvae (L1) (17 h after egg eclosion) were used for BBMV preparation. Due to the minute size of larval thrips, 100−200 mg of L1 bodies (over one thousand thrips) were pooled from four different cohorts in the span of one month and stored at −80°C. Pooling insects into one biological sample necessitated the achievement of enough material for BBMV preparation and one total protein isolation for proteomic analysis. BBMVs were prepared by MgCl_2_ precipitation and differential centrifugation according to [19] with modifications. Thrips were homogenized in 3 mL of MET-PI buffer (300 mM mannitol, 5mM EGTA, 17 mM Tris-HCL pH 7.5), supplemented with 1X Halt™ Protease and Phosphatase Inhibitor Single-Use Cocktail (Thermo Fisher Scientific, Waltham, MA, USA) with the aid of a Dounce homogenizer (20 strokes in ice). After centrifugation at 1,000 ✕ g for 10 min at 4°C, thrips homogenate was subjected to precipitation with MgCl_2_ by adding 1/19 volume of 240 mM MgCl_2_ and incubating in ice for 15 min. Then, the mixture was centrifuged at low speed (2,500 ✕ g for 10 min 4°C) resulting in a pink-colored pellet. The pellet was homogenized with MET buffer and an additional MgCl_2_-precipitation was done followed by a second low-speed centrifugation (2,000 ✕ g for 10 min at 4°C) which resulted in a white pellet and a white supernatant. Then, the supernatant was ultracentrifuged at 300,000 ✕ g for 1.5 h at 4°C. The resulting pellet was resuspended in 2 mL of MET buffer and re-homogenized using a Dounce homogenizer. An aliquot of this pellet was collected and named here as “semipurified”. After another precipitation with MgCl_2_ and ultracentrifugation (300,000 ✕ g for 1.5 h at 4°C), the resulting pellet containing the BBMVs was resuspended in 50 µL of MET-PI buffer. Total protein was quantified using Bradford method using bovine albumin serum as standard [23]. We used two different approaches to validate our method of BBMV isolation: (i) enrichment of aminopeptidases through enzymatic assay; and (ii) separation of BBMV proteins by SDS-PAGE and visualization of band enrichments in silver-stained gels. To assess the enrichment of aminopeptidases, we tested the activity of these enzymes by quantifying the fluorescence emitted after hydrolyzation of the leucine from the fluorescent probe using the Leucine Aminopeptidase (LAP) Activity Assay Kit (Abcam, Cambridge, United Kingdom). While the substrate in this kit (L-leucine-7-amido-4-methylcoumarin) measures the activity of LAP, it also serves as a substrate to aminopeptidase N (APN), an abundant insect gut epithelium membrane protein. To compare the enrichment at each step of the purification, i.e., fractions include homogenate, semipurified, and BBMVs, 1 µg of protein was separated by electrophoresis in a 13% (w/v) polyacrylamide gel. The resulting protein bands were stained with Pierce ™ Silver Stain for Mass Spectrometry (Thermo Fisher Scientific, Waltham, MA, USA) and photographed using the iBright CL1000 imager (Thermo Fisher Scientific, Waltham, MA USA).

### Qualitative analysis of BBMV proteins

#### Nano liquid chromatography tandem mass spectrometry (nano LC-MS/MS).

The single BBMV sample was submitted to the Duke Proteomics and Metabolomics Core Facility for protein extraction, digestion and mass spectrometry. The sample was resuspended in a solution containing 8 M urea and then sonicated using an ultrasonic homogenizer at a frequency of 20–25 Khz for two to five, 10–30 s bursts. The resulting protein sample was split into two aliquots to serve as two technical replicates (i.e., subsamples) for all steps in the following protocol. The subsamples were prepared for LC-MS/MS analysis using the S-Trap™ microspin column digestion protocol (PROTIFI). This involved adding 20% (w/v) SDS to the subsamples to achieve final concentrations of 3% (w/v), reducing them with Tris(2-carboxyethyl) phosphine, alkylating them with methyl methanethiosulfonate, acidifying them with phosphoric acid, and loading them onto S-traps for trypsin digestion. The resulting proteins were eluted from the column and lyophilized to dryness. To perform qualitative LC-MS/MS, each subsample was resuspended in a solution containing 1% (v/v) trifluoroacetic acid and 2% (v/v) acetonitrile, supplemented with yeast alcohol dehydrogenase, and then loaded onto a column. Four microliters (5%) of each subsample was used for the LC-MS/MS analysis, which was carried out using a nanoAcquity Ultra Performance Liquid Chromatography system from Waters Corp, coupled to a Thermo Orbitrap Fusion Lumos high-resolution accurate mass tandem mass spectrometer (Thermo Fisher Scientific, Waltham, MA, USA), via a nanoelectrospray ionization source and a FAIMS pro Duo interface, which increased the number of proteins detected.

### Protein identification and prediction of gut brush border membrane proteins

Using the Mascot search engine (v.2.5.1), proteins present in the BBMV subsamples were identified by searching the peptide spectra against a database of the *F. occidentalis* genome-derived proteome (Focc_OGS.pep.v1.1, [24]), with carbamidomethyl as the fixed modification and oxidation as the variable modification. Trypsin was specified as the proteolytic enzyme, with a minimum of two missed cleavages. Scaffold viewer (v.4.9.0) was used to perform a qualitative analysis of the proteins, and the confidence of protein identification was determined based on the number of exclusive unique peptides per protein. The “exclusive unique spectrum count” was used to identify proteins that were unique or similar between the subsamples. The BBMV proteins were annotated using OmicsBox v2.1.14 [25].

Cellular location of BBMV proteins was predicted similarly as described by Tavares [19]. Briefly, amino acid sequences of the identified proteins underwent cell location predictions based on available software: Cello2GO [26], BUSCA [27], Deeploc [28], GPI-SOM [29] and PredGPI [30].

Only proteins predicted to be associated with the cell-membrane or secreted were retained in the final, non-redundant protein dataset between the two proteomics subsamples. Next, these proteins were curated against the UniProt database using the “reviewed” Uniprot database FASTA and annotations files (uniprot_sprot.fasta/dat – both downloaded in August 2023). *Frankliniella occidentalis* sequence data were retrieved from the associated amino acid FASTA file (Focc_OGSv1.1_pep.fa). All BLASTp queries were conducted against the Swiss-Prot protein database (uniprot_sprot.fasta). A custom Python function (extract_subcellular_locations) (S2 File) was implemented to extract cell location data from the UniProt DAT file. The function specifically searched for lines in the Swiss-Prot protein database starting with “CC -!- SUBCELLULAR LOCATION:”. The cell locations were filtered based on a pre-defined list of words (“Cell membrane”, “Secreted”, “Extracellular”, “Apical cell membrane”, “Extracellular matrix”). For each protein identifier, the corresponding protein sequence was fetched from the FASTA file. These sequences were then subjected to BLASTp analysis against the Swiss-Prot database. BLASTp was configured to use the BLOSUM62 matrix with an E-value cutoff of 10e-5 [25]. Lastly, to be included in the final data set each protein had to satisfy all of the following criteria: i) BLASTp e-value less than or equal to 10e-5; ii) at least two unique peptides identified the protein as determined by the LC-MS/MS analysis; iii) the protein was identified in both BBMV subsamples. Peptides with the same “FOCC” identifiers were treated as a single entity to calculate the normalized spectral abundance factor for the identified protein. We are naming this dataset as ‘brush border membrane proteome’, predicted to include membrane proteins, proteins of both outer and inner plasma membrane leaflets and secreted proteins.

To assess the relative abundance of proteins on the gut brush border membrane, the Distributed Normalized Spectral Abundance Factor (dNSAF) method was used on the brush border membrane proteins dataset [31]. Briefly, the dNSAF is a quantitative metric used in proteomics to determine the relative abundance of proteins in a complex sample. The dNSAF method provides a normalized measure that accounts for both the number of spectra matching a particular protein and the length of that protein, offering a more accurate representation of protein abundance. In the present study, the dNSAF values were calculated for each protein identified in both BBMV subsamples, and the mean dNSAF value (n = 2) was calculated for each protein. Using JMP (Student Edition 18) statistical software (JMP Statistical Discovery LLC, Cary, NC), a histogram analysis on the data indicated a non-normal distribution pattern (exponential) and performing a generalized regression analysis using the maximum likelihood estimation method to fit the data, exponential distribution was determined to be the best model fit (AIC = −5177.4). To compare the relative abundance of proteins between the three cell locations of significance to brush border membranes - “apical cell membrane”, “cell membrane”, and “secreted”, a generalized linear mixed model (GLIMMIX) analysis was performed on the mean dNSAF dataset using exponential distribution (link function = Log) followed by pairwise comparisons of least square mean estimates between cell locations (Tukey HD).

## Results

### Validation of BBMV enrichment

**Fig 1:**
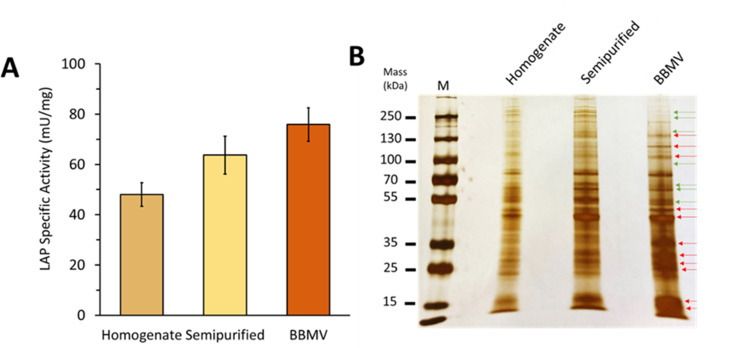
Enrichment of *Frankliniella occidentalis* gut brush border membrane proteins. **(A)** Aminopeptidase-specific activity (leucine amino peptidase and aminopeptidase N) in fractions from whole thrips first instar (L1), 17 h-synchronized larvae; bars represent the mean of three subsamples from each fraction of the BBMV purification step + /- standard deviation. **(B)** SDS–PAGE profiles of the BBMV fractions at each step. BBMV preparations were separated by SDS–PAGE on a 13% (w/v) acrylamide gel and silver-stained. Red arrows represent enriched bands and green arrows are bands that were removed in the enrichment. M: PageRuler prestained protein marker. One microgram of total protein was loaded in each lane.

As thrips homogenates progressed through the enrichment procedure, we observed a more than 1.5-fold increase in aminopeptidase activity from the starting material (Fig 1A), an indication of BBMV isolation and enrichment. Resolution and staining of proteins on an SDS-PAGE showed enrichment of proteins of less than a molecular mass of 55 kDa in the BBMV-isolated samples compared to the semipurified and homogenate samples (Fig 1B), confirming enrichment of BBMV.

### Proteins identified and predicted to localize to the brush border membrane of first instar larvae (L1) of *Frankliniella occidentalis*

The LC-MS/MS proteomic analysis of two technical replicates of BBMV-isolated proteins (subsamples) identified 2544 proteins in common (Table S1 in S3 File) after removal of contaminants, internal standards, and sample-processing proteins (trypsin, alcohol dehydrogenase, albumin, casein, and keratin). Notably, more than 15 proteins were identified as aminopeptidases further lending support to successful BBMV isolation from whole bodies. From the total set of BBMV proteins, in silico sequence prediction analyses were performed sequentially (Table 1) to filter the list by features that infer cell location and secretion, and to provisionally annotate thrips proteins as cell membrane or secreted proteins based on identity to homologs of known cell membrane or secreted roles, resulting in a final set of 469 proteins with a putative designation of “brush border membrane proteome”, including proteins from both plasma membrane inner and outer membrane leaflet as well as secreted proteins (Table S3 in S3 File). Protein localization predictions placed cell membrane-associated proteins as most represented in the dataset (42%), followed by secreted proteins (34%) and glycosylphosphatidylinositol anchor signal (GPI) or a signal peptide (SP)-containing protein (24%). Sixty-six of these proteins were predicted to have features indicating all three of these subcellular localization patterns.

**Table 1:** Manually applied bioinformatics pipeline for identifying putative brush border membrane (BBM) proteins and number of proteins meeting criteria at each step.

Selection Step (in order of operation)	Number of Proteins
1.LC-MS/MS-identified proteins shared between the two BBMV^a^ replicates	2544^b^
2.Predicted to have cellular locations using multiple localization prediction tools^c^	1333^d^
3.Nonredundant proteins^e^ predicted to be associated with the cell membrane or secreted	960
4.Matched UniProt^f^ homologs annotated as cell membrane or secreted proteins^g^	790
5.Brush border membrane proteome: E-value ≤ 1e-5 in the previous step (4), found in both replicates, ≥ 2 unique peptides identified by LC-MS/MS for that protein, and removal of proteins with redundant identifiers.	469

^a^ BBMV = brush border membrane vesicles isolated from pools of whole bodies of first instar larvae (L1) of *Frankliniella occidentalis.*

^b^ after removal of contaminants, internal standards, and sample-processing proteins.

^c^ predictions made with Cello2GO, BUSCA, Deeploc, GIPSOM, PredGPI and Phobius.

^d^ Number of proteins predicted to be extracellular = 459, cell membrane-associated = 559, and glycosylphosphatidylinositol anchor signal (GPI) or a signal peptide (SP)-containing = 315; proteins with all three predictions = 66.

^e^ removal of duplicate occurrences between proteomics subsamples.

^f^ UniProt “reviewed” database proteins. Searched using keywords for cell location category: “cell membrane”, “secreted”, “extracellular”, “apical cell membrane” and “extracellular matrix”.

^g^ Refer to Table S2 in S3 File for list of annotated thrips proteins classified as “extracellular”, “cell membrane”, “apical cell membrane” or “secreted”.

### Majority of BBM proteome proteins match the larval gut proteome

**Fig 2:**
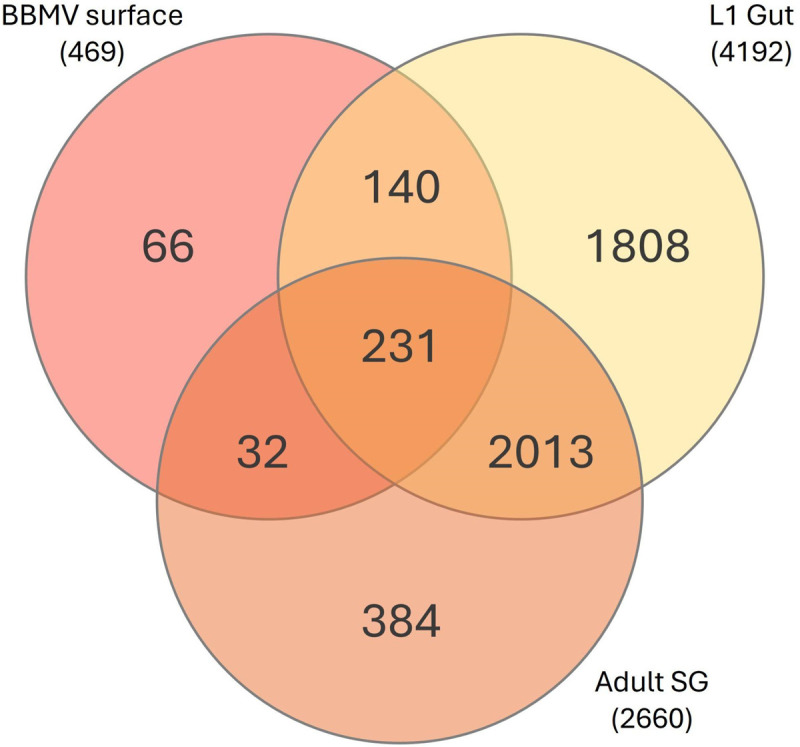
Overlap of tissue-derived proteomes with the BBM proteome. The *Frankliniella occidentalis* official gene set (v1.1) database of protein identifiers (FOCC IDs) was used to compare first-instar larval (L1) BBM proteins identifiers and annotations with two published tissue proteomes of L1 guts [21] and adult salivary glands [32] of the same isolate of *F. occidentalis* used in this study. The numbers in parentheses indicate the total number of proteins identified and annotated for each system.

Pairwise comparisons of the resulting brush border membrane proteome (469 proteins) to a previously reported first-instar larval gut proteome, (4192 proteins [21]) and adult salivary gland (SG) proteome (2660 proteins [32]) of *F. occidentalis* (Table S4 in S3 File) revealed shared and tissue-specific proteins among the three proteomes (Fig 2). Of the brush border membrane proteins, 79.1% were exclusively identified in the larval gut proteome, whereas 6.8% were exclusively identified in the adult SGs. There were also 66 proteins unique to the BBM proteome in our comparisons. These BBM-unique proteins might be explained by low abundance or difficult-to-extract proteins not captured in the gut or SG tissue proteome analyses, developmental stage-dependent expression of these proteins in larval and absent in adult SG tissues, and/or occurrence of these proteins in BBMs of Malpighian tubules. Nonetheless, the majority of the brush border membrane proteome aligned with the larval gut. The complete list of shared and tissue-specific proteins with their annotations is provided in Table S3 in S3 File.

### Relative abundance of BBM proteins

**Fig 3:**
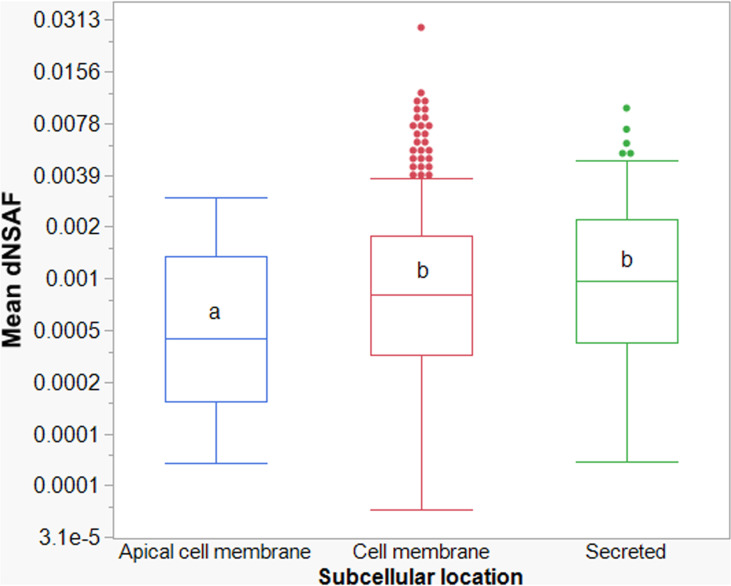
Variation in distributed normalized spectral abundance factors (dNSAF) of *Frankliniella occidentalis* first instar larval (L1) brush border membrane proteins predicted to be localized to the apical cell membrane, cell membrane, or secreted. For better visualization of the variation in the dataset, mean dNSAF values were plotted on a log_2_ scale (y-axis). Box and whiskers plots display the distribution of the dNSAF values around the median (midpoint line), with box ends marking the lower quartile (Q1, 25%) and upper quartile (Q3, 75%) of the values, respectively, and whisker ends marking the minimum/ maximum values for each subcellular location group: apical membrane (n = 27 proteins), cell membrane (n = 275 proteins), secreted (n = 167 proteins); the dots represent outlying values in the dataset. Generalized linear mixed model analysis (GLIMMIX) was performed on the mean dNSAF values (n = two subsamples per protein) using exponential distribution (link function = Log) followed by pairwise comparisons between cellular location groups (Tukey HD) using least square mean estimates. Letters in common indicate no difference between pairs. Refer to Table S3 in S3 File for the dNSAF dataset.

Based on dNSAF values, the mean abundance of the predicted cell membrane proteins (n = 275) and secreted proteins (n = 167) was comparable (Fig 3) (*P* = 0.8153). However, the mean abundance of proteins predicted to be localized to the apical membrane (n = 27) was significantly lower than those predicted to be localized in the cell membrane (*P* = 0.0045) or secreted (*P* = 0.0147).

### Relative abundance of the BBMV proteins by gene ontologies and inferred molecular functions

**Fig 4:**
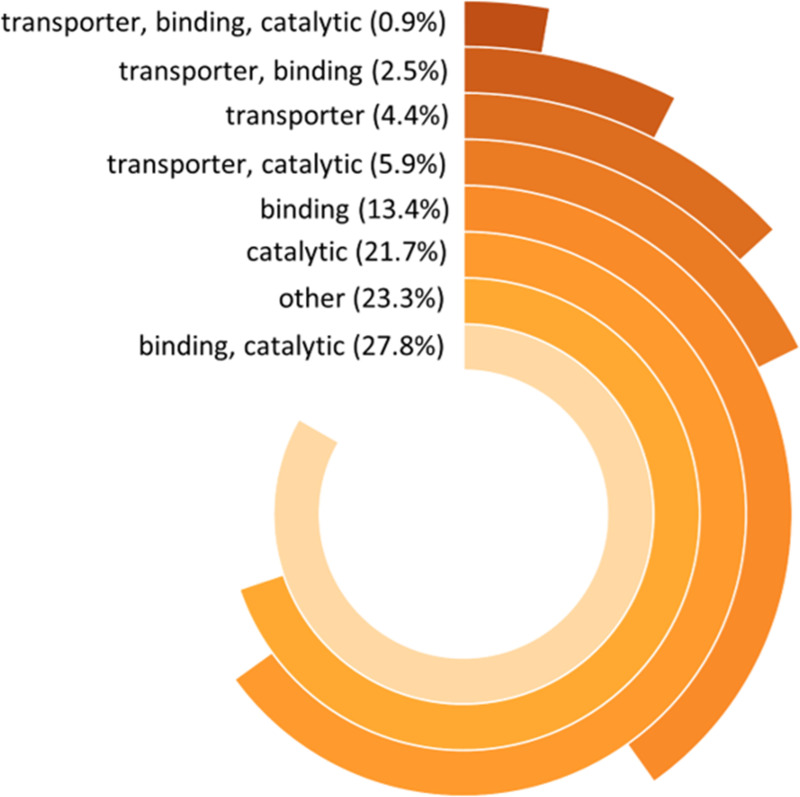
Relative contributions (%) of molecular-function gene ontology (MOL GO) categories to the abundance of brush border membrane proteins. Molecular function predictions of *Frankliniella occidentalis* BBM proteins were determined from first instar larvae (L1) using BLAST2GO, and the Distributed Normalized Spectral Abundance Factor (dNSAF) was calculated for each protein. Percent of BBM protein abundance attributed to each MOL GO category (seven in total) = Σ (mean dNSAF value for proteins classified within a category) ∕ Σ (mean dNSAF value for proteins in all categories).

Approximately 98% of the BBMV proteins had BLASTx matches to known proteins – the remaining were annotated as hypothetical proteins – and ~96% (459) were assigned molecular function GO terms. Overrepresented functions were either solely ‘catalytic activity’ (119), ‘binding’ (59), or ‘transporter activity’ (34) or multi-functional (i.e., seven MOL GO categories) for any given protein (121). Proteins with no MOL GO or no GO assignment (136) were classified as ‘other’ (Tables S3 in S3 File). Average dNSAF values calculated for each protein were used to determine the relative protein abundance (%) contributed by proteins classified into each MOL GO category to total protein abundance across all MOL GO categories (Fig 4). Proteins assigned to both binding’ and ‘catalytic activity’ were the most abundant at the BBM (27.8%), followed by ‘other’ (23.3%). Proteins assigned only one MOL GO category, either binding (13.4%) or catalytic activity (21.7%), were also major contributors to protein abundance in the BBM. Proteins with provisional transporter activity alone or in combination with the other MOL GO categories accounted for an estimated 4.4% and 9.3%, respectively, of the BBM protein abundance.

**Fig 5:**
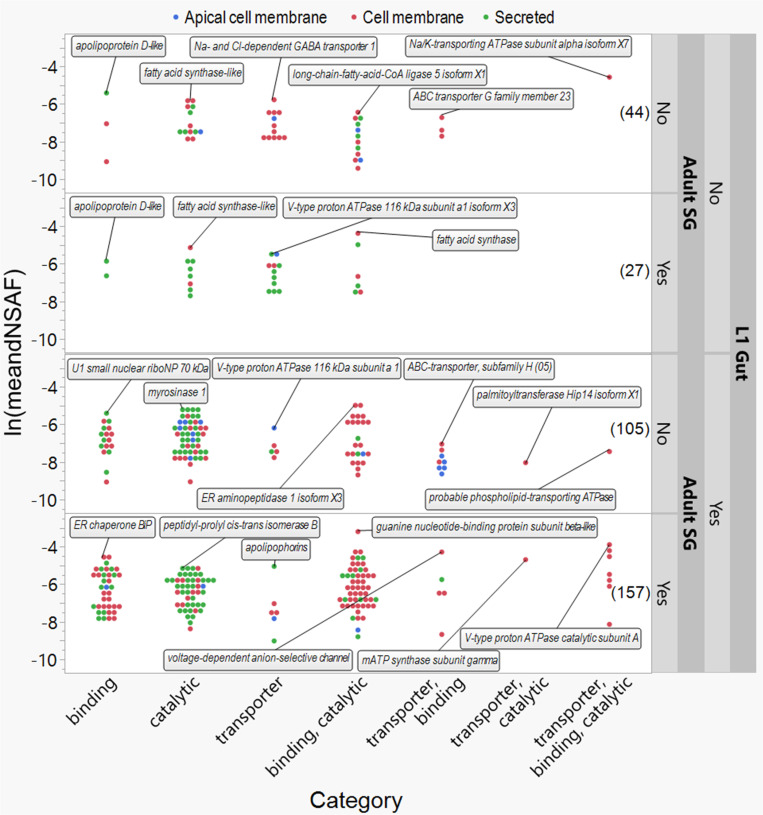
Two-way distribution of identified proteins of the epithelial brush border membrane (BBM) of first instar larvae (L1) of *Frankliniella occidentalis* by abundance [Distributed Normalized Spectral Abundance Factor (dNSAF)] and gene ontology (GO) terms (Categories) that infer single and multiple molecular functions in the cell. These GO-annotated proteins, predicted to be associated with the apical cell membrane, cell membrane, or secreted at the BBM, are displayed by tissue type [L1 gut and adult salivary glands (SG)] as determined by comparisons with two published *F. occidentalis* tissue proteomes [21,32]. Numbers in parentheses indicate the number of GO-annotated BBM proteins identified in neither tissue, adult SG alone, L1 gut alone, or both tissues. Boxed text and line indicate BLASTx-annotated proteins of known function that were determined to have the highest abundance in the BBM within each GO category. Putative BBM proteins with no GO-annotation or no molecular GO categories (136) are not depicted here. Refer to Table S3 in S3 File for a complete list of GO-annotated BBM proteins and their corresponding dNSAF values.

To further discriminate BBM proteins of the gut from salivary glands, we examined protein occurrence, identities, and abundance (dNSAF) in relation to the seven MOL GO categories (Fig 5). Proteins with the highest relative abundance within their assigned GO category and having putative homologs to proteins with known functions are indicated in Fig 5.

Cell-membrane proteins were the most abundant across the seven MOL GO categories (17 proteins), of which six were found in both guts and SG proteomes. One of those proteins, ‘guanine nucleotide-binding protein subunit beta-like protein,’ was determined to be the most abundant protein in the entire BBM proteome dataset, a protein of which homologs are involved in the recruitment, assembly and/or regulation of a variety of signaling molecules. The other five were ‘V-type proton ATPase catalytic subunit A’, ‘guanine nucleotide-binding protein G(o) subunit alpha’, ‘Hexamerin’, ‘voltage-dependent anion-selective channel’, and ‘ATP synthase subunit beta, mitochondrial’. Unique to larval guts, ‘U1 small nuclear ribonucleoprotein 70 kDa’, ‘myrosinase 1’, ‘V-type proton ATPase subunit a1’, ‘endoplasmic reticulum aminopeptidase 1 isoform X3’, ‘ABC transporter-H subunit’, ‘palmitoyltransferase Hip14 isoform X1’ and ‘phospholipid-transporting ATPase IA isoform X4‘ had the highest abundance in their GO categories. Among the 27 proteins found only in salivary gland tissues, the most abundant proteins within their MOL GO categories were two fatty acid synthases (cell membrane-associated), isoform X3 of ‘V-type proton ATPase subunit a1’, and ‘apolipoprotein D-like’. Unique to the BBMV dataset are ‘apolipoprotein D-like’, ‘fatty acid synthase-like’, ‘sodium- and chloride-dependent GABA transporter 1’, ‘long-chain-fatty-acid--CoA ligase 5 isoform X1’, ‘ABC transporter G family member 32’ and ‘sodium/potassium-transporting ATPase subunit alpha isoform X7’, the most abundant proteins within their MOL GO category.

Across the brush border membranes of BBMV, SG, and gut, our analysis points to a significant overlap and unique distribution of proteins involved in catalytic activity, binding, and transporter functions. Many proteins with catalytic and binding activities are shared across all three datasets, reflecting their roles in core biological processes such as metabolism and cellular signaling. Conversely, transporter proteins demonstrate a more distinct distribution with considerable numbers uniquely present in the BBMV and gut proteomes. BBMV proteins with catalytic activity and binding shared with the gut proteome are mostly involved in carbohydrate, protein, and lipid transformations, displaying extensive binding capacities to various molecules, including GTP, calcium, and RNA. In terms of transporter activity, the gut brush border membrane proteome is characterized by a significant presence of cell membrane proteins, particularly those with ABC-type transporter activity and sodium/potassium transporting capabilities. These proteins are crucial for regulating cellular transport of substances like ions and lipids.

## Discussion

In this study, we present a comprehensive analysis of the protein composition and levels on the midgut brush border membrane of thrips larvae. A novel procedure was established to isolate brush border membrane vesicles (BBMVs) from the entire body of first instar *F. occidentalis*. Previous studies with other insect species have typically focused on isolating BBMVs from gut tissues, whereas our method utilizes the whole organism. Given the small size of the larvae (0.4–1 mm in length), a substantial amount of material, 100–200 mg (~1 mL of larvae), was required to successfully isolate BBMVs. Although our findings are based on one pooled biological sample of larval thrips, the sample represented the BBMV proteome from multiple, independently sampled cohorts (over 1,000 individuals). Using liquid chromatography-tandem mass spectrometry (LC-MS/MS), we identified the proteins in the BBMV samples. The localization of these proteins on the gut brush border membrane was determined using a combination of bioinformatics prediction tools and verification against reviewed protein databases. Comparison between previously published gut and adult salivary gland proteomes allowed the prediction of proteins residing on the brush border membrane of those tissue systems, a pioneering effort in the study and understanding of thrips’ gut physiology and its interactions at the molecular level.

As expected, proteins known for their association with cellular membranes were enriched in the thrips BBMV. Proteins involved in binding activities are prominently located in the cell membrane. This positioning is essential for their role in facilitating cell-to-cell communication and molecular recognition, which are critical for processes like hormone signaling, nutrient uptake, and immune responses. Enzymes with catalytic functions are predominantly associated with the cell membrane, including the apical membrane, pointing to their role in metabolic processes that are directly linked to the cellular interface. This strategic localization supports their involvement in key metabolic pathways that require rapid response to external stimuli and efficient substrate handling. Proteins with transporter activity are overwhelmingly associated with the membrane regions which reflects their role in controlling the passage of molecules across cellular barriers. This includes ion channels, nutrient transporters, and efflux systems, all of which maintain cellular homeostasis by regulating the internal concentration of various biochemicals [33].

### Similarities across the three proteomes

Our analyses revealed substantial overlaps in proteins identified in the whole body-isolated BBMVs, gut and salivary gland tissues of *F. occidentalis*. More cell membrane and secreted proteins were exclusively shared between the BBMV and gut as compared to the SGs, likely due to the surface area and size of the thrips gut relative to the salivary gland tissue systems [34], and the number of proteins representing the two tissue proteomes used as subject databases. Another possible explanation may include that the same thrips developmental stage (L1) used in the present study was used to generate the available gut proteome database, while the only available proteome for thrips SGs was generated from *F. occidentalis* adults.

### BBM proteome of *Frankliniella occidentalis* and other insects

Variation in the proportion of brush border membrane proteins was observed between insect species whose BBMV proteome has been elucidated. In *F. occidentalis* (this study), approximately 18.4% of the total identified proteins were predicted to localize in the cell membrane and secreted. This is higher compared to *Diaphorina citri* surface proteome where only 7.4% and 7.1% of proteins were predicted in adults and nymphs, respectively. *Trichoplusia ni* showed 6.2% of proteins predicted to the cell surface [15]. Proteomics of head and salivary gland tissues of *Aedes aegypti* identified 3790 proteins [14], but no further analysis was done to identify surface proteins. One possible reason for the difference is that those studies considered only proteins in the outer plasma membrane leaflet while this study considers both plasma membrane leaflets as well as secreted proteins. If this work was to consider only the surface proteins confirmed by homologs in the UniProt database, only 42 out of 960 (4.38%) proteins are located to the outer plasma membrane. The methodological approach, particularly the use of computational analysis, in this work, versus manual curation [19] might also influence these findings. These disparities likely reflect the distinct physiological and dietary adaptations of each species. *F. occidentalis*, for example, are adapted to a broad range of plant materials and their proteome likely facilitates the processing of various plant substances, indicating a versatile digestive system. Additionally, each insect must deal with different pathogens and stressors that would affect the adaptations found in the proteome. Additionally, in this work, BBMVs were isolated from thrips whole body whereas in Tavares [19], BBMVs were isolated from dissected guts, which is likely to influence the number of proteins identified in this study as BBMVs from salivary gland and malpighian tubules could contribute to the protein diversity.

### Proteomics of BBMV isolated from thrips whole body provide insights into thrips digestion

Thrips midguts are likely to be acidic. A gut pH of 3–5 is reported for adults of *F. occidentalis* [35,36] but the pH of larval stages for this species has not been measured. A pH of 5–6 has been reported for adults and larva of *Thrips tabaci* and *Thrips imaginis* [37]. The presence of α-amylase, trypsin, and tryptase activity has been reported for whole bodies of adults and larvae of *F. occidentalis* and it changes depending on the diet, life stage, and generation [38]. Larvae of *F. occidentalis,* for example, have more enzyme activity when compared to adults, which could be explained by their active feeding behavior [38]. Hindering activity of gut proteases by feeding *F. occidentalis* with protease inhibitors impairs larval development suggesting that amino acids are essential for the success of this developmental stage [36,39].

Among the gut brush border membrane proteins identified with provisional catalytic activity, several are reported to be involved in the metabolism of proteins, carbohydrates, and lipids. In this study, the majority of gut proteases were secreted, with a large diversity of serine peptidases over cysteine peptidases. Plants, the major source of nutrients for herbivorous insects, are rich sources of sugar, however very limited in nitrogen content. To surmount this nutritional challenge, insects evolved a sophisticated system to break down and absorb peptides, the main nitrogen form available in a plant-based diet. Plants, on the other hand, selected an arsenal of protease inhibitors to impair insect development compromising the activity of insect proteases and consequently nitrogen absorption. Thrips mainly feed on plant tissue, which can contain protease inhibitors as part of the plant’s defense mechanisms. Plants often produce specific inhibitors against a wide range of proteases, including cysteine proteases [40]. Contrary to cysteine proteases, serine proteases may be less affected by these inhibitors or have evolved to resist them, giving thrips an advantage in digesting plant proteins efficiently. In this work, BBMVs were isolated from a colony of thrips that has been feeding on green beans for many generations. The most common protease inhibitors in legumes, like green beans, are the Bowman-Birk inhibitors (BBIs) and the Kunitz-type inhibitors, which are known to inhibit serine proteases such as trypsin and chymotrypsin [40–42]. The high diversity of serine proteases over cysteine proteases in the thrip’s gut BBM despite their diet of green beans with protease inhibitors could suggest that thrips may produce serine proteases in excess to saturate the inhibitors present. Over time, thrips might have selected for or adapted serine proteases that are less sensitive to inhibition by the specific protease inhibitors produced in green beans. The evolutionary pressure exerted by the ingestion of protease inhibitors might have led to an increase in the secretion of serine proteases as a compensatory mechanism, supported by other digestive adaptations [43]. A similar compensatory strategy has been documented for the interaction of Colorado Potato Beetle and potato plants [44,45] as well as aphids (*Myzus persicae*) and *Nicotiana benthamiana* plants [46]. Additionally, thrips might have developed methods of deactivating or digesting the inhibitors post-ingestion before they can affect the proteases, such as by pH changes in the gut, by other enzyme actions [47], or through gut microflora partnerships [48]. The presence of different isoforms of serine proteases, which vary in their sensitivity to inhibitors, can also account for a variety of functional proteases in the gut. Some isoforms may be inhibitor-resistant due to conformational changes that protect critical active sites [47].

Besides plants, *F. occidentalis* has diverse feeding habits ranging from plant sap, pollen, fungi, and even other insects. The varied diet likely necessitates a digestive system that can function effectively under different pH conditions. Serine proteases, which are known to operate well across a range of pH levels, are particularly suited to this task, more so than cysteine proteases which are less adaptable [5]. This versatility in serine proteases may have been evolutionarily advantageous, allowing thrips to efficiently process a wide variety of food sources by maintaining an optimal environment within their gut for enzyme activity. This adaptability supports the hypothesis that the thrips’ gut environment has evolved to maximize their digestive capability across their diverse dietary habits. Given the abundance of serine proteases, cysteine proteases may serve as a complementary system, engaged when specific substrates are present or when serine proteases are inhibited by plant-derived or other exogenous protease inhibitors [47].

In this study, carbohydrate-degrading enzymes were less abundant and diverse than the proteases in the larval BBM, reinforcing the idea that amino acids are important for the development of thrips larvae. The prevalence of serine proteases has also been observed in the gut of larval Lepidoptera and the midguts of Orthoptera and Diptera [49]. Carbohydrate digestion is supported by the secretion of proteins involved in the degradation of oligo- and disaccharides into monosaccharides as well as plant complex carbohydrates, e.g., cellulose, as seen by several beta-glucosidases on the gut BBM. A few other proteins acting on carbohydrates are enzymes that play a role in the maintenance of the extracellular matrix, especially acting on O-glycosylated compounds.

In most insects, dietary lipids are less crucial than proteins and carbohydrates. This is because insects can synthesize many fatty acids and phospholipids from dietary carbohydrates. However, insects need dietary sources of sterols and polyunsaturated fatty acids for cell membrane structure, secondary metabolites, and as precursors for steroid synthesis [49]. The abundance of lipid transporter proteins like apolipoproteins, ‘Niemann-Pick-C-1’ protein, and phospholipid transporting ATPases indicates a specialized adaptation for lipid handling. The scarcity of lipases in the gut brush border membrane, combined with the abundance of lipid transporters, could suggest that lipid digestion may not be the primary limiting step in lipid utilization for thrips. Alternatively, it might indicate that lipases are present but not among the most abundant proteins, or lipid breakdown may occur extracellularly or in specific gut regions not represented in the protein sample. It’s also possible that thrips might rely more on direct absorption of certain lipids from their diet [49–51] or utilize symbiotic relationships with gut microbes for lipid processing [52–54].

V-type Proton ATPases were represented in the list of proteins with ‘transporter activity’. They are pivotal in acidifying various compartments, including the gut lumen. This acidification can aid in digestion by activating certain digestive enzymes and providing an optimal pH for their function, which is crucial for the breaking down of complex macromolecules. Additionally, the gut pH can influence the composition and activities of the gut microbiota, which in turn can affect nutrient processing and the overall health of the insect. This has been seen in *F. occidentalis* when silencing V-ATPase-B by injecting double-stranded RNA (dsRNA) into adults led to a marked increase in mortality and a decrease in fertility among the treated thrips. Silencing of the gene also negatively affected the survival rate and production of fewer viable offspring [55].

The data also revealed other significant processes associated with proteins annotated with binding activity. The thrips’ ability to process signals from diverse stressors is facilitated by proteins like calcineurin B homologous protein, vital for cellular communication and regulation. Oxidoreductases and peroxiredoxins form part of the thrips’ proteins for an effective oxidative stress response important in defending against metabolic byproducts and environmental challenges.

### Signal transduction, energy metabolism, and cellular interactions

GTPases were the most frequent GO Name in the list of *F. occidentalis* proteins with binding and catalytic activity and included ras-related proteins and guanine nucleotide-binding proteins, key regulators of signal transduction pathways, playing vital roles in processes such as cell growth, differentiation, and nutrient sensing [56,57]. The presence of these proteins suggests a dynamic regulatory environment on the gut brush border membrane, capable of rapidly responding to various internal and external stimuli. The presence of proteins with kinase activity, particularly those involved in ATP binding, points to active phosphorylation processes, which are crucial for a wide array of cellular functions, including protein synthesis and modification. This could indicate a high level of protein turnover and processing in the thrips gut, necessary for maintaining gut integrity and function. The presence of enzymes like Fatty Acid Synthase, with multiple catalytic activities related to fatty acid biosynthesis, aligns with the previously noted importance of lipid metabolism in thrips. These enzymes suggest a significant investment in energy storage and membrane synthesis, essential for the gut cells’ structural integrity and metabolic activities [58,59]. Proteins like superoxide dismutase and other oxidoreductases highlight the gut’s role in managing oxidative stress. These enzymes are critical for neutralizing reactive oxygen species, protecting gut cells from damage that can result from metabolic processes and environmental stressors [60,61].

The protein profile on the thrips gut brush border membrane, as indicated by the abundance and variety of ATP-binding proteins and ATP synthase components, highlights a significant focus on energy metabolism and ion transport. The presence of various ATPases, especially those involved in proton and calcium transport, indicates active regulation of ion homeostasis. This is crucial for maintaining the gut environment, affecting everything from nutrient absorption to enzyme activity [62,63]. Sodium/potassium-transporting ATPase and calcium-transporting ATPase, in particular, play essential roles in maintaining electrochemical gradients across cell membranes, which are fundamental for cellular functions in the gut [64]. ABC transporters, which are well-represented in the list, are key players in transporting a wide range of substances, including metabolic products, lipids, and xenobiotics. Their presence suggests mechanisms for detoxification and managing the chemical composition of the gut environment. This might be particularly relevant in the context of dietary specialization, as thrips feeding on specific plant materials like green beans might encounter various phytochemicals that require efficient transport and detoxification systems [21,55,65].

### Gut brush border membrane proteins and pathogen exploitation

#### Proteins identified in this study previously reported as TSWV Gn interactors.

*F. occidentalis* is considered a “supervector” that efficiently transmits TSWV and several other plant pathogenic microbes [2,66]. Attachment is one of the first steps of virus entry which has been reported to rely on the interaction of viral proteins with several host surface proteins and several pieces of evidence point out the role of the glycoprotein N (Gn) as the TSWV attachment protein in the thrips gut [67–69].

Although complex, a few studies have investigated proteins that behave as molecular interactors of TSWV Gn and that might play a role in virus entry. By separating the proteins of *F. occidentalis* in a 2D gel electrophoresis associated with membrane overlay assay with purified TSWV virions or recombinant Gn glycoprotein, [70] identified six thrips proteins that interact with the tomato spotted wilt virus (TSWV) attachment protein, Gn. These include Cuticular protein-V (CP-V; GenBank accession QBP34368.1), Endocuticle structural glycoprotein-GN (endoCP-GN; GenBank accession QBP34367.1), Endocuticle structural glycoprotein-V (endoCP-V; GenBank accession QBP34366.1), Cyclophilin (peptidyl-prolyl cis-trans isomerase; GenBank accession QBP34370.1), Enolase (GenBank accession QBP34369.1) and Mitochondrial ATP synthase α-subunit (GenBank accession QBP34371.1). Out of these six proteins, two, endocuticle structural glycoprotein (endoCP-GN) and cyclophilin were found to directly interact with the TSWV Gn protein in insect cells. Here, only one protein of the six interactors was found in the brush border membrane, the ATP synthase subunit alpha, mitochondrial, the second most abundant protein in the brush border membrane proteome (Mean dNSAF 0.016373), found in gut, salivary gland, and saliva proteome and the BBM proteome of both organs. CypA reported by [70] was not identified in the brush border membrane proteome of the present study, however, the proteomic methods varied between the two studies and this may influence the outcome. Nonetheless, CypB (peptidyl-prolyl cis-trans isomerase B) was identified in the present study, a protein with the same activity and domain as CypA [56.25% identical (Evalue = 4x10^-65) and 71% query coverage). The thrips CypB was predicted to be secreted in all three datasets (Mean dNSAF 0.005893). Alternatively, CypA may play a role in the viral replication cycle after virus entry.

#### Proteins identified in this study that need further investigation of their potential role as viral interactors.

Unlike aphids and whiteflies, that transmit a wide variety of plant viruses, thrips appear to be specialized to transmit viruses in the *Orthotospovirus* (circulative, propagative transmission), *Ilarvirus*, *Carmovirus*, *Sobemovirus* and *Machlomovirus* genera [71]. The binding partners and in some cases the receptors of these non-thysanopteran insect transmitting viruses have been characterized [20], but little is known about the receptor or binding partners of thrips-transmitted viruses beyond TSWV.

In this study, several proteins were annotated with an endocytosis or receptor function, the two molecular functions of proteins associated with virus entry. The Scavenger Receptor Class B Member 1 (SR-B1), particularly the isoform X2, is a significant receptor for some viruses, notably hepatitis C virus (HCV). SR-B1 is primarily involved in cholesterol metabolism but also facilitates HCV entry into hepatocytes. The interaction between HCV envelope glycoproteins and SR-B1 is crucial for the virus’s attachment and entry, making SR-B1 a co-receptor in this process. This was demonstrated in a study where blocking SR-B1 significantly reduced HCV infection, showing its role in the viral life cycle [72–74]. Adaptor Protein complex 2 (AP-2; FOCC006777-PA-CDS) is reported as involved in clathrin-mediated endocytosis, a pathway often explored by viruses for its role in cargo recognition and vesicle formation, which are important process in the internalization of viruses during infection [75]. G protein-coupled receptors (GPCRs; FOCC003578-PA-CDS, FOCC011250-PA-CDS, FOCC012085-PA-CDS, FOCC003577-PA-CDS, and FOCC001156-PA-CDS) and arrestins (FOCC013190-PA-CDS) are integral to the entry mechanisms of various viruses. GPCRs mediate responses to external stimuli and undergo conformational changes upon activation, facilitating interactions with arrestins, which regulate GPCR activity. Arrestins not only desensitize GPCRs, aiding in their internalization, but also redirect signaling pathways to G-protein-independent routes, crucial for the internalization and trafficking of the receptor-virus complex into host cells [76].

### Gut brush border membrane proteins and insect control

One of the novel strategies for controlling thrips involves the use of genetically engineered (GE) crops that express specific *Bacillus thuringiensis* (Bt) pesticidal proteins. For instance, the modified bacterial pesticidal protein (BPP) Mpp51Aa1 (Cry51Aa2.834_16), expressed in MON 88702 cotton, has shown effectiveness against *Frankliniella fusca* and *F. occidentalis* [77]. This BPP suppressed oviposition (egg laying) by thrips, with varying effects on the species tested. It also caused developmental delays in larvae, indicating its potential as a novel mechanism for pest control in Bt crop plants. The proteome of brush border membrane vesicles (BBMVs) plays a pivotal role in identifying putative interactors and defining the mode of action of pesticidal proteins. By analyzing the proteome of BBMVs, it is possible to identify specific receptors or binding sites for pesticidal proteins, which is crucial in understanding how these toxins exert their lethal effects on pests such as thrips. Homologs of known pesticidal protein interactors were found in thrips brush border membrane (this study), including aminopeptidase-N and ATP-binding cassette proteins (ABC proteins) [78]. This knowledge is instrumental in elucidating the mode of action at a molecular level, revealing how toxins disrupt critical physiological processes in the insect gut. Furthermore, the identification of these interactors aids in the development of more efficient and targeted insecticidal strategies. It also provides insights into potential resistance mechanisms, as changes in these membrane proteins could lead to reduced binding and efficacy of the toxins. Overall, the BBMV proteome serves as a fundamental tool in advancing our understanding of insect-pathogen interactions, paving the way for innovative approaches in pest management and contributing to sustainable agricultural practices.

## Supporting information

S1 File _Raw_images. (PDF)

S2 File Python script for extracting subcellular locations from a uniprot reviewed database. (TXT)

S3 File **S1 Table. Scaffold analysis and proteins detected from BBMV of whole body *F. occidentalis*. S2 Table. BLASTp result of BBM proteins searched in Swiss-Prot database containing reviewed proteins.** 790 proteins were retrieved from a BLAST search conducted on the Swiss-Prot database (curated with reviewed proteins of which subcellular location has been addressed) using amino acid sequences from 960 *F. occidentalis* nonredundant proteins predicted to be associated with the cell membrane or secreted. **S3 Table. Western Flower Thrips (*Frankliniella occidentalis*) BBM proteome final dataset.** 790 *F. occidentalis* proteins obtained from BLAST search (S2 Table). Proteins with E-value cutoff equal or lower than 10e-5 were filtered to compose the BBMproteome comprising 469 proteins. dNSAF: Distributed Normalized Spectral Abundance Factor. Top 10 most abundant proteins are highlighted. **S4 Table. List of protein IDs (FOCC ID) for gut and salivary gland proteome datasets.**
(XLSX)
