# Supplementary material for: Protein landscape of the brush border membrane of first instar larvae of Frankliniella occidentalis, the western flower thrips
Source: PLoS One. 2025 Jun 24;20(6):e0326260. doi: 10.1371/journal.pone.0326260 (PMC12186882; doi:10.1371/journal.pone.0326260)
Supplement: S1 File — (PDF) [file pone.0326260.s001.pdf]

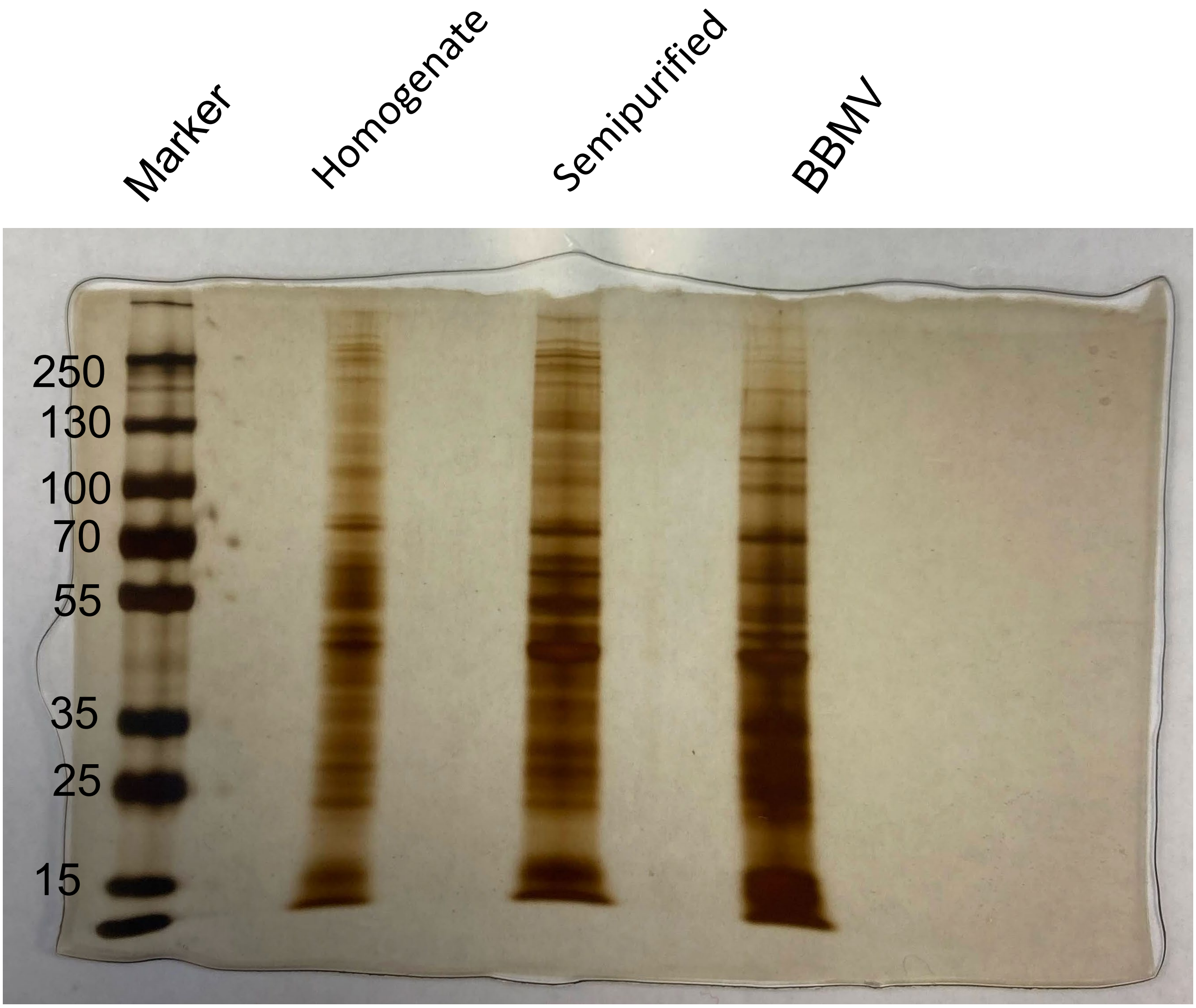

Figure 1B. SDS–PAGE profile of BBMV isolated from *Frankliniella occidentalis* using whole first instar larvae. BBMV preparations were separated by SDS–PAGE on a 13% (w/v) acrylamide gel and silver-stained. Red arrows represent enriched bands and green arrows are bands that were removed in the enrichment. Marker: PageRuler prestained protein marker. One microgram of total protein was loaded in each lane.
